# Visual outcomes in tuberculum sellae meningiomas comparing transcranial and endoscopic endonasal approaches

**DOI:** 10.1016/j.wnsx.2024.100319

**Published:** 2024-03-08

**Authors:** Ricardo Marian-Magaña, Marcos V. Sangrador-Deitos, Luis Rodríguez-Hernández, Jorge A. Lara-Olivas, Germán López-Valencia, Rodolfo Villalobos-Díaz, Jorge F. Aragón-Arreola, Karen E. Padilla-Leal, Jesús Humberto García-Zazueta, Alfredo Camacho-Castro, Juan L. Gómez-Amador

**Affiliations:** a Department of Neurosurgery, National Institute of Neurology and Neurosurgery “Manuel Velasco Suárez”, Av. Insurgentes Sur 3877, Tlalpan, 14269, Mexico City, Mexico; b Department of Neurosurgery, Culiacan General Hospital Dr Bernard J Gastelum, Av. Juan Aldama s/n Esquina Calle Estado de Nayarit Col. Gral. Antonio Rosales, Culiacán, Mexico

**Keywords:** Endoscopic endonasal approaches, Meningioma, Transcranial approaches, Tuberculum sellae, Visual outcomes

## Abstract

**Background:**

Tuberculum sellae meningiomas (TSM) account for 3–10% of intracranial meningiomas. Visual loss is the presenting symptom in up to 80% of cases. Surgical management poses a great challenge due to tumor proximity to neurovascular structures such as the optic nerve and the internal carotid artery (ICA); hence, there is controversy regarding the optimal approach. The aim of this study is to determine differences in visual outcomes between transcranial (TCA) and endoscopic endonasal (EEA) approaches.

**Methods:**

A retrospective study including 29 patients with TSM surgically treated by TCA or EEA between 2011 and 2023 in a single referral center was conducted. Pre-and post-operative neuro-ophthalmologic evaluations, focusing on visual acuity and campimetry, were evaluated.

**Results:**

Sixteen (55.16%) patients were intervened through a TCA and the remaining 13 (44.84%) via an EEA. The lesions in each group were similar in terms of pre- operative volume (15.12 vs 12.9 cm^3^, p = 0.497) and neurovascular invasion (optic canal invasion 48.26 vs 41.37%, p = 0.664; ICA 44.81 vs 31.03%, p = 0.797). There were no significant differences in visual outcomes between both approaches; TCA presented an improvement of 5.18 points in visual fields (p = 0.140), whereas EEA had an improvement of 17.39 points in visual acuity (p = 0.114).

**Conclusion:**

EEA seems to offer greater improvement in visual acuity than TCA. However, the ideal approach should be individualized; taking into account the tumor’s volume and invasiveness, as well as the patient's visual complaints.

## 1 Introduction

Meningiomas, which originate from the arachnoid cap cells, are the most common intracranial extrinsic tumors, representing 24–30% of all CNS primary tumors.^1^ In most cases, meningiomas are sporadic. However, there are genetic disorders such as neurofibromatosis type 2) that increase their incidence. Environmental factors such as radiation exposure and trauma also pose a risk for the development of these neoplasms in susceptible patients.^2^

Tuberculum sellae meningiomas (TSM) originate from the optic groove, optic chiasm sulcus, and tuberculum sellae region.^3^ They account for 3–10% of intracranial meningiomas. The average age at diagnosis is 52 years, with a 2:1 female-to-male ratio. The most common presenting symptom is visual loss in up to 80% of cases. Impairment of visual acuity is present in 75% of cases, and visual field abnormalities in 80%, along with headache in 35%.^4^ Other less common symptoms include facial paresthesia, cognitive alterations, seizures, endocrinopathies, and anosmia, which may suggest alternative diagnoses.

These tumors pose a great challenge due to their proximity to the optic nerve, internal carotid artery (ICA), anterior cerebral artery (ACA), hypothalamus, infundibulum, and pituitary gland. They typically lie in a suprasellar or sub-chiasmatic position, displacing the optic chiasm postero-superiorly and the optic nerves laterally.^5^ Visual deficit is the primary indication for surgical management, and radiation therapy is occasionally used for asymptomatic patients. Meningiomas should be resected early in patients with visual symptoms, emphasizing an accurate preoperative assessment for subsequent objective surgical evaluation.

There is considerable controversy regarding the optimal surgical approach. Some authors advocate for the transcranial approaches (TCA) using a variety of corridors, or endoscopic endonasal approaches (EEA).^6^ Classically, TCA have been the gold standard for management and good visual outcomes have been reported.^7^, ^8^, ^9^ EEA are a valid option in appropriately selected patients.^10^^,^^11^

This study aims to determine any significant differences regarding visual outcomes when comparing the TCA and EEA in patients with TSM. We also present representative cases and discuss the role of each approach.

## 2 Methods

### 2.1 Patient population and data collection

**Fig. 1:**
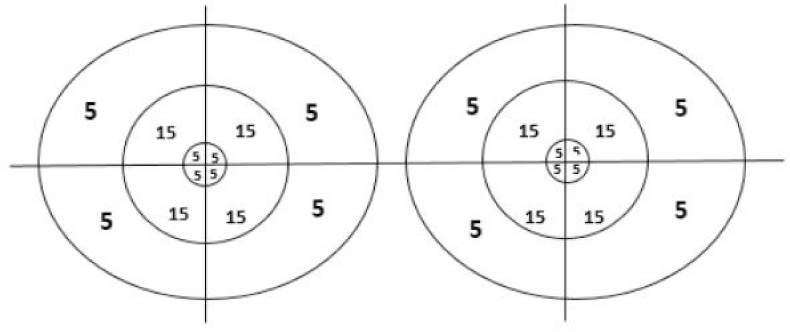
Representation of the visual field deficit score. A total score of 100 represents total blindness.

We conducted a descriptive, longitudinal, and retrospective study of patients with the diagnosis of TSM that were surgically treated by TCA or EEA in a single tertiary referral center over a 12-year period (between 2011 and 2023). The institutional board review approved this protocol; our institution's Ethics Committee did not require patient consent due to its retrospective design. Patients with incomplete clinical charts, younger than 18 years, without confirmed pathological diagnosis, or without pre and postoperative magnetic resonance imaging (MRI) or pre and postoperative neuro-ophthalmology evaluations were excluded. Clinical records were reviewed for the preoperative and postoperative neuro-ophthalmologic evaluation, focusing on visual acuity and campimetry; both parameters were analyzed based on the visual impairment score (VIS), as described by Fahlbush and Schott.^4^ In this scale, a score of 100 and 50, for visual acuity and campimetry, respectively, represent a blind patient, and a score of 0 represents a patient with no visual affection. Campimetric defects were also analyzed by using the visual field deficit (VFD) score (Fig. 1). Tumoral volume measurements were assessed through volumetric analysis using Elements SmartBrush software (Brainlab, Munich, Germany); these were calculated based on pre and postoperative (3 months) contrast-enhanced MRI.

### 2.2 Statistical analysis

Data are presented as means and standard deviations for continuous variables, and as percentages for categorical variables. The Shapiro–Wilk test was used to determine distribution normality. Differences between categorical variables were determined with a chi-squared test, whereas, for mean comparison of continuous variables, T and Wilcoxon tests were used, according to data distribution. A *p*-value of <0.05 was considered statistically significant. Data were analyzed using STATA 17.

## 3 Results

### 3.1 Clinical and radiological variables

A total of 29 patients were analyzed, being 26 (89.7%) females, with a mean (SD) age of 50.68 (13.21) (range 33–83). As for the surgical approach chosen, 16 (55.16%) patients were intervened via a TCA and 13 (44.84%) via an EEA. The preoperative tumor volumes for both groups did not significantly differ (*p* = 0.497), with a mean volume of 15.12 cm**^3^** for the TCA group and 12.9 cm**^3^** for the EEA group. No significant differences were found in the postoperative volumes (*p* = 0.283), as well. Also, no differences regarding optic canal invasion (*p* = 0.664) or ICA/ACA involvement (*p* = 0.797) were found. These similarities between groups demonstrate that there was no selection bias towards any of the approaches, and similar lesions were included in both (Table 1).

**Table 1:** Demographic and operative characteristics.

Variable	TCA (*n* = 16)	EEA (*n* = 13)	*p*-value
**CLINICAL VARIABLES**			
**Gender – n (%)**			
- Male	2 (6.89)	1 (3.44)	0.672
- Female	14 (48.27)	12 (41.37)	
**Mean age – mean (S.D.)**	56.56 (13.73)	43.46 (8.33)	0.271
**RADIOLOGICAL VARIABLES**			
**Mean tumor volume**			
- Mean preop. vol. in cm^3^ – mean (S.D.)	15.12 (9.18)	12.9 (7.93)	0.497
- Postop. vol. in cm^3^ – mean (S.D)	0.545 (1.33)	1.26 (2.16)	0.283
**Optic canal invasion – n (%)**			
- None	2 (6.89)	1 (3.44)	0.664
- Unilateral	7 (24.13)	4 (13.79)	
- Bilateral	7 (24.13	8 (27.58)	
**ICA/ACA involvement –n (%)**			
- Contact	3 (10.34)	4 (13.79)	0.797
- Partially encases	6 (20.68)	4 (13.79)	
- Completely encases	7 (24.13)	5 (17.24)	

ACA: anterior cerebral artery; EEA: endoscopic endonasal approach; ICA: internal carotid artery; Postop.: postoperative; Preop.: preoperative; TCA: transcranial approach.

### 3.2 Visual outcomes

Patients were grouped by type of intervention and categorized based on the difference between pre and postoperative VIS. Regarding visual acuity, the TCA approach presented a worsening of 4.31 points (*p* = 0.565) and the EEA presented an improvement of 17.39 points (*p* = 0.114); as for visual fields, the TCA approach had an improvement of 5.18 points (*p* = 0.140) and the EEA a worsening of 1.47 points (*p* = 0.658) (Table 2). No significant differences between both surgical interventions were found. For this analysis, improvement or deterioration in visual acuity and visual fields, were determined by any change in Snellen visual acuity tests and VFD score. In the TCA group, regarding visual acuity, 3 (10.34%) patients remained unchanged, 2 (6.89%) improved, and 6 (20.68%) worsened in both eyes, with 3 improving and 1 worsening in only one eye, and lastly, one patient presented improvement in one eye and worsening in the other. As for visual fields, 8 (27.58%) remained unchanged, while 6 (20.68%) worsened in both eyes, with the remaining 2 patients in this group presenting improvement in one eye with worsening of the other. In the EEA group, regarding visual fields, 2 (6.89%) remained unchanged and 3 (10.34%) improved, with none worsening, in both eyes, while 6 (20.68%) improved and 2 worsened in one eye. As for visual fields, 5 (17.24%) remained unchanged and 4 (13.79%) worsened in both eyes, with 2 improving and 2 worsening in only one eye. Of the 29 patients included 5 (17.24%) remained unchanged, 15 (51.72%) presented any degree of improvement, and 9 (31.03%) presented any degree of worsening in visual acuity; 13 (44.82%) remained unchanged, 4 (13.79%) presented any degree of improvement, and 12 (41.37%%) presented any degree of worsening in visual fields defects (Table 3).

**Table 2:** Visual outcomes.

Variable	TCA (*n* = 16)	EEA (*n* = 13)	*p*-value
**PREOPERATIVE EVALUATION**			
**Visual acuity VIS – mean (S.D.)**	49.31 (34.90)	74.15 (30.67)	0.054
**Visual fields VIS – mean (S.D.)**	13.93 (12.11)	19.76 (15.13)	0.258
**Papilledema – n (%)**	3 (10.34)	1 (3.44)	0.606
**POSTOPERATIVE EVALUATION**			
**Visual acuity VIS – mean (S.D.)**	53.62 (31.25)	56.76 (33.26)	0.795
**Visual fields VIS – mean (S.D.)**	8.75 (9.27)	21.23 (14.23)	**0.008**
**Papilledema – n (%)**	3 (10.34)	1 (3.44)	0.606
**Δ in visual functions**			
**Visual acuity VIS (***p***-value)**	+4.31 (0.565)	−17.39 (0.114)	
**Visual fields VIS (***p***-value)**	−5.18 (0.140)	+1.47 (0.658)	

EEA:endoscopic endonasal approach; TCA: transcranial approach; VIS: Visual Impairment Scale; Δ: change; (+): worsening; (−) means: improvement.

**Table 3:** Visual functions and surgical details of 29 patients.

Case	Tumor volume (cm^3^)	_Snellen visual acuity_				_VFD score_			
		_Preop_		_Postop_		_Preop_		_Postop_	
		Rt	Lt	Rt	Lt	Rt	Lt	Rt	Lt
1	22.8	HM	20/400	HM	20/400	80	80	80	80
2	12.8	NLP	20/100	NLP	20/40	100	20	100	20
3	11.9	20/400	LP	20/400	NLP	0	75	50	100
4	14.8	20/400	20/800	20/100	20/30	20	20	50	50
5	5.7	20/400	NLP	20/50	NLP	80	100	80	100
6	33.4	LP	20/25	HM	HM	50	50	75	75
7	3.8	20/30	20/40	20/20	20/20	0	0	0	20
8	12.1	HM	20/400	HM	20/30	80	75	80	50
9	4.6	20/200	20/200	20/100	HM	50	50	50	50
10	10.9	NLP	20/25	NLP	20/40	100	50	100	80
11	8.4	HM	20/400	20/50	20/100	95	80	95	80
12	11.7	HM	20/40	NLP	20/20	80	75	80	0
13	14.7	NLP	HM	NLP	HM	100	95	100	95
14	12.4	LP	20/30	HM	20/20	50	50	75	0
15	33.4	20/30	LP	20/25	NLP	75	80	20	100
16	19	NLP	NLP	NLP	NLP	100	100	100	100
17	21.6	20/25	20/25	NLP	20/80	50	50	100	80
18	7.2	HM	NLP	20/50	NLP	50	100	50	100
19	26.4	LP	20/40	HM	20/40	95	0	95	0
20	24.1	LP	20/400	NLP	NLP	95	50	100	100
21	4.8	20/20	20/20	20/40	20/40	0	0	15	15
22	12.3	20/150	20/25	20/60	20/25	25	25	25	25
23	6.5	20/400	20/30	20/400	20/30	20	20	20	20
24	25.8	HM	LP	NLP	NLP	80	95	100	100
25	20.1	20/25	HM	NLP	NLP	50	20	100	100
26	5.4	20/40	20/30	20/80	20/60	20	20	20	20
27	7.1	20/60	20/40	20/60	20/40	0	0	0	0
28	8.9	20/80	20/200	20/20	NLP	50	50	80	100
29	16.6	HM	20/125	20/200	20/80	50	0	50	0

HM: hand motion; LP: light perception; Lt: left; NLP: no light perception; Rt: right; VFD: visual field deficit.

*An EEA approach was performed from patients 1–13; a TCA was performed in patients 14–29.

### 3.3 Representative cases

#### 3.3.1 Case 1

**Fig. 2:**
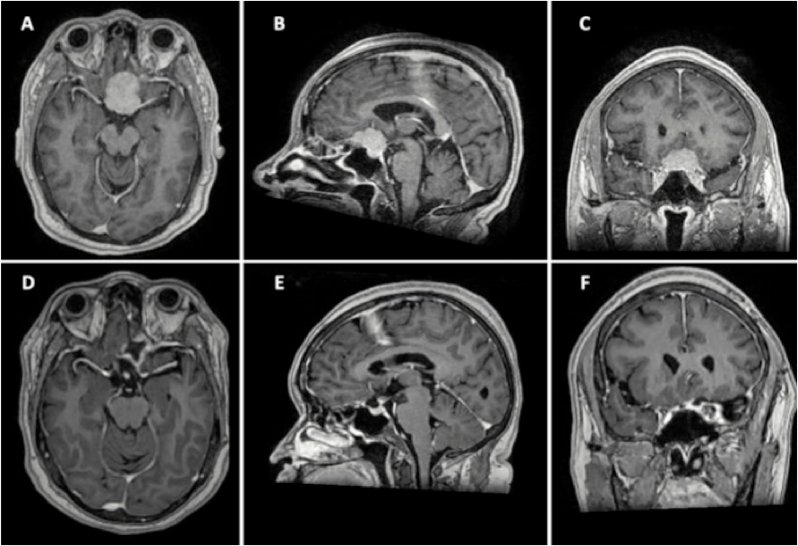
**(A**–**C) Preoperative MRI:** from left to right: axial, sagittal, and coronal views from a T1WI C+ showed an extra-axial lesion on the tuberculum sellae region, with suprasellar extension, and partial involvement of the left ICA. **(D**–**F) Postoperative MRI:** from left to right: axial, sagittal, and coronal views from a T1WI C+ in which gross total resection is observed.

**Fig. 3:**
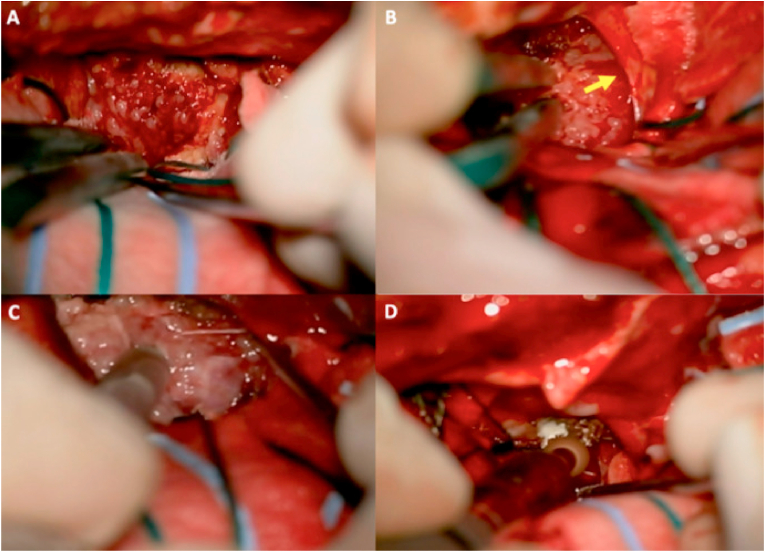
**Intraoperative images of case 1. (A)** Initially, tumor debulking was performed with bipolar coagulation and suction. **(B)** Careful dissection of the right optic nerve *(yellow arrow)* is completed. **(C)** Ultrasonic aspiration was used for debulking of the fibrous part of the tumor **(D)** After completing the resection, the sella tubercle was drilled. (For interpretation of the references to colour in this figure legend, the reader is referred to the web version of this article.)

A 53-year-old female with no relevant medical background presented with a 2-month history of visual loss in the left eye and episodic headaches. Neurological examination revealed bitemporal hemianopsia with visual acuity of 20/80 in the right eye and 20/200 in the left eye. Contrast-enhanced MRI revealed an extra-axial lesion with homogeneous enhancement located in the tuberculum sellae region with extension to the anterior cranial fossa compatible with a TSM (Fig. 2). Resection of the lesion through a left lateral supraorbital approach was decided, achieving gross total resection (Fig. 3). The patient was discharged 5 days later with visual improvement in the right eye and visual impairment in the left eye.

#### 3.3.2 Case 2

**Fig. 4:**
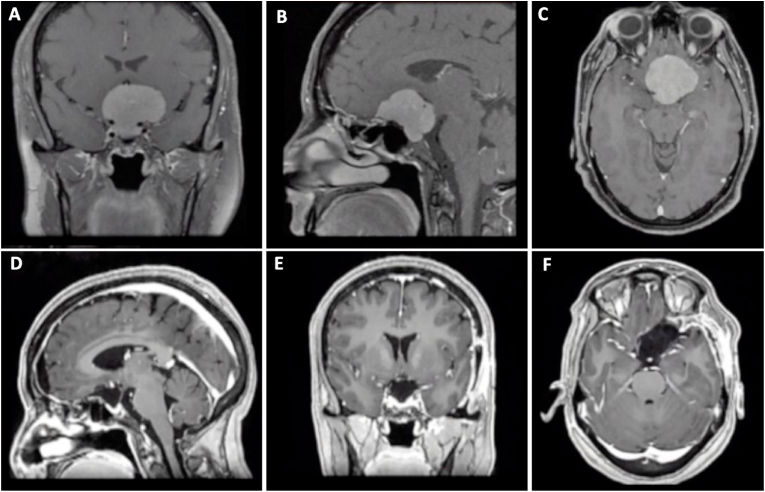
**(A**–**C) Preoperative MRI:** from left to right: coronal, sagittal, and axial views from a T1WI C+ showed an extra-axial lesion on the tuberculum sellae region, with suprasellar extension which obliterates the floor of the third ventricle, and an asymmetric growth over the dorsal portion of the left ICA. Encasement of both ICA can be observed. **(D**–**F) Postoperative MRI:** from left to right: axial, coronal, and axial views from a T1WI C+ in which gross total resection is observed.

**Fig. 5:**
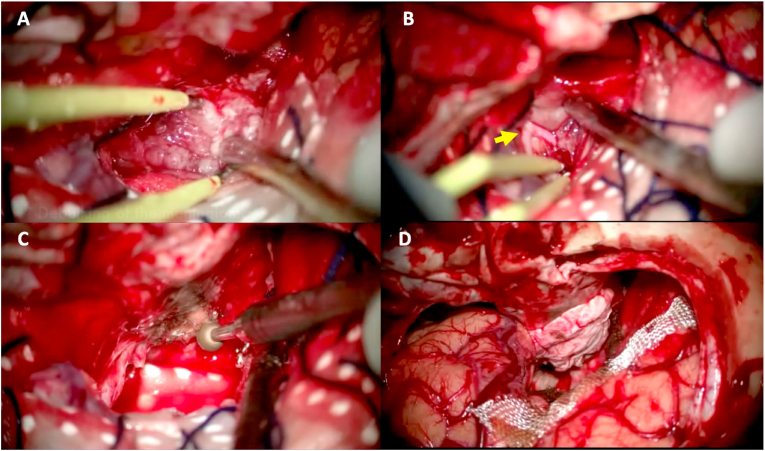
**Intraoperative images of case 2. (A)** After performing the surgical approach and dural opening, the lesion along with its implant, were located along the tuberculum sellae and planum sphenoidale region. Bipolar coagulation of the dural implant was initially performed. **(B)** Dissection of the left A1 segment of the ACA *(yellow arrow)* was performed in order to proceed with a safe resection. **(C)** After gross total resection, the tuberculum sellae was drilled. **(D)** A panoramic view of the surgical field showed gross total resection with no other structural disturbances. Dural reconstruction was performed using an autologous pericranial patch. (For interpretation of the references to colour in this figure legend, the reader is referred to the web version of this article.)

A 57-year-old man with no relevant medical background presented with a history of 6 months of bilateral visual loss, predominantly in the left eye. Neurological examination revealed a visual acuity of 20/800 in the left eye and 20/400 in the right eye with a temporal campimetric defect. Contrast-enhanced MRI revealed an extra-axial lesion with homogeneous enhancement in the tuberculum sellae which presented suprasellar extension towards the anterior recess of the third ventricle with an asymmetric portion protruding dorsally over the left ICA. Extension over the planum sphenoidal was also present (Fig. 4). A left pterional approach was performed, and gross total resection was achieved (Fig. 5). No postoperative visual improvement was attained.

#### 3.3.3 Case 3

**Fig. 6:**
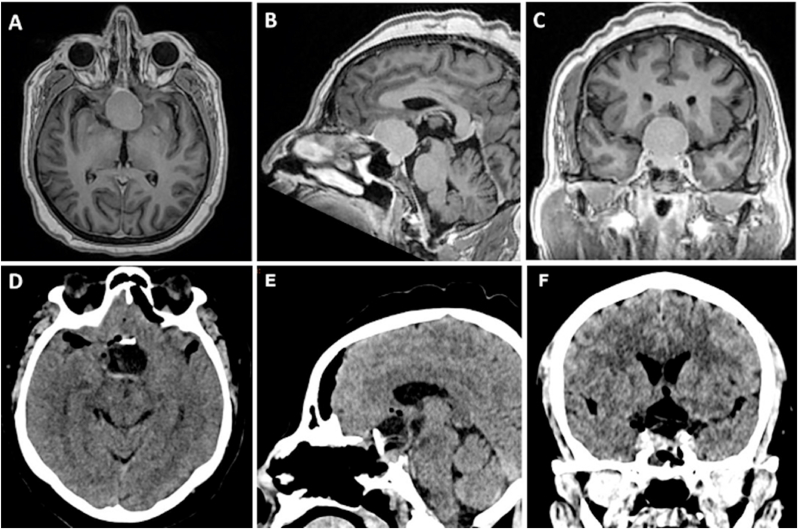
**Preoperative MRI: (A)** axial, **(B)** sagittal, and **(C)** coronal views from a T1WI C+ showed a dorsally projected lesion compatible with a TSM. **Postoperative CT scan: (D)** axial, **(E)** sagittal, and **(F)** coronal views in which gross total resection is observed.

A 50 year-old female with one-year complaints of headache and bilateral loss of visual acuity, was referred to our hospital. Neurological examination revealed a visual acuity of 20/25 in the left eye and 20/150 in the right eye. Brain MRI revealed a lesion located within the suprasellar space compatible with a TSM (Fig. 6). The patient underwent resection of the lesion via an endoscopic endonasal extended transplanum-transtuberculum approach. Postoperative CT showed no residual tumor and the patient was discharged from the hospital on the sixth postoperative day. At follow-up, no changes in visual function were found.

#### 3.3.4 Case 4

**Fig. 7:**
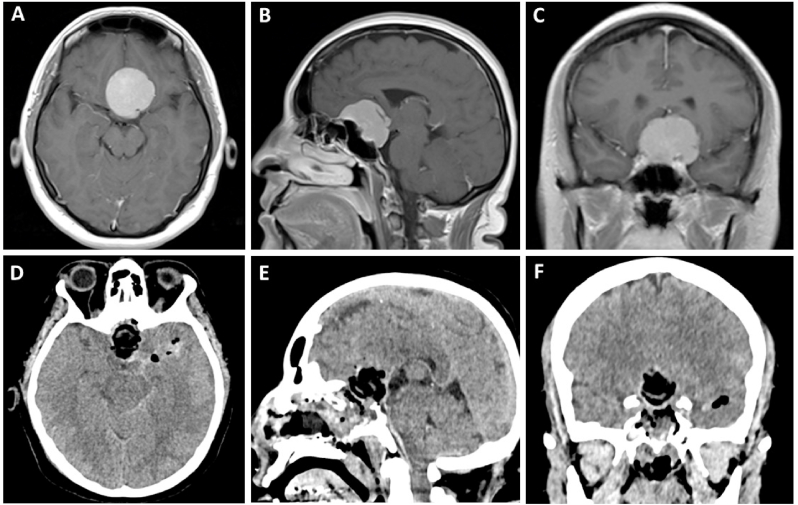
**Preoperative MRI: (A)** axial, **(B)** sagittal, and **(C)** coronal views from a T1WI C+ revealed an homogeneous enhancing solid sellar lesion, originating from the tuberculum sellae with extension to the sellar region and upper third of the clivus. **Postoperative CT scan: (D)** axial, **(E)** sagittal, and **(F)** coronal views in which gross total resection is observed.

A 41 year-old female with an unremarkable medical background, was referred to our hospital due to complaints of insidious visual loss in both eyes, as well as abulia and memory impairment. A formal visual examination was performed, revealing a visual acuity of 20/400 in the left eye and hand motion in the right eye. Campimetry was relevant for temporal hemianopsia on the left eye. Brain MRI revealed a vividly enhancing sellar mass originating from the tuberculum sellae (Fig. 7). An extended endoscopic transplanum-transtuberculum-transclivus approach was performed for tumor resection. No postoperative CSF leak was observed postoperatively and the patient was discharged on day 7 with visual improvement in the left eye at 6 months follow-up.

## 4 Discussion

Choosing the best surgical approach for TSM has been a debated topic because of the multiple approaches available for resection. Nowadays, there is no consensus of which approach is the standard of care and selection is usually based on the tumor volume. For small tumors with no vascular encasement or optic canal invasion, neurosurgeons prefer EEA, and for the ones presenting with lateral growth, artery encasement, optic canal invasion, or third ventricular invasion, the transcranial route is preferred.^12^^,^^13^

TCA have been classically considered the standard of treatment since they provide effective citorreduction and visual improvement. Several approaches for TSM have been employed including unilateral or bilateral subfrontal, frontotemporal, and interhemispheric approaches.^14^, ^15^, ^16^ However, its invasiveness, the requirement of a craniotomy, and visible skin incisions, pose notable disadvantages. On the other hand, EEA have gained popularity due to its reduced invasiveness and potential for early patient recovery, thus, becoming an alternative to TCA.^17^, ^18^, ^19^

A relevant advantage of the EEA is that they provide direct access to the meningioma's attachment, allowing for early devascularization. Additionally, the use of the endoscope allows the neurosurgeon to achieve a better exocranial visualization of the surrounding para-sellar anatomy (i.e. the medial and inferior portions of the optic canal), less manipulation of the optic nerves, and less olfactory nerve and brain retraction.^20^^,^^21^ During early development of EEA, surgeons performed wide approaches to the tuberculum sellae region, which usually needed complex reconstruction techniques and a higher rate of CSF leaks was present. Before the popularization of the pedicled nasoseptal flap technique, this led to many surgeons opting to avoid EEA.^22^ Once novel reconstruction techniques and materials were discovered, the rate of CSF leaks has reduced dramatically.^23^, ^24^, ^25^

Several studies have compared the results between these two surgical approaches to evaluate their efficacy and determine which is more favorable in terms of visual preservation.^12^^,^^14^^,^^20^^,^^21^^,^^26^^,^^27^ Some authors have suggested that EEA may offer faster and sustained improvement compared to TCA. This is primarily attributed to the less invasive nature of EEA, which allows a faster recovery.^14^^,^^21^ Bander et al compared EEA with TCA in meningiomas of the tuberculum sellae and the planum sphenoidale. They found similar results in terms of complete resection, as well as better visual results, less brain retraction, and fewer seizures with EEA.^21^ Kitano et al analyzed the surgical outcomes of TCA and expanded transsphenoidal surgery (ETSS) in 28 patients with TSM. They found a significant improvement in visual acuity (*p* = 0.010) in the ETSS group, with no significant improvement in visual fields between the two groups. The main advantages of the ETSS are the direct exposure of the inferomedial surface of the optic canal allowing for direct visualization and early decompression of the optic nerves.^14^ Song et al found that both approaches had similar results in terms of visual improvement and complete resection rates. However, EEA showed lower rates of postoperative complications and shorter hospital stay compared to TCA.^26^

A systematic review by Turel et al revealed comparable rates of resection, low complication rates and significant visual improvement in both approaches.^20^

Furthermore, Jimenez et al conducted a systematic review on approaches for suprasellar meningiomas. While they did not specifically focus on TSM, its findings indicated that both surgical approaches were similarly effective in terms of resection rates and visual improvement. However, EEA showed advantages in terms of lower complication rates and shorter recovery time when compared to TCA.^28^ Koutourousiou et al evaluated EEA for suprasellar meningiomas, including those involving the tuberculum sellae. The study showed promising results in terms of visual improvement and complete resection using the endoscopic technique.^29^ Kong et al highlighted the importance of considering specific anatomical characteristics when choosing the appropriate surgical approach. While it does not directly focus on visual outcomes, it underscores the significance of proper surgical approach selection to optimize outcomes.^30^

All these results are supported by the systematic review and meta-analysis on endoscopic surgery for TSM by Clark et al.^10^

There are predictors of surgical success that should be considered when choosing a surgical approach. Some authors have demonstrated that preoperative visual acuity is a predictor of postoperative outcome.^31^^,^^32^ Age is also a predictive factor, with poorer outcomes observed in older patients. Patients presenting within 6 months of symptom appearance are more likely to recover, compared to those presenting with symptoms lasting over 1 year.^33^ Kshettry et al described the anatomical and pathological factors to determine the optimal surgical approach, considering as absolute indications for TCA: lateral extension that surpasses the optic nerves, ACA encasement, and kissing carotids; meanwhile, for EEA, a sellar extension with prefixed optic chiasm was considered as an absolute indication.^13^

Regarding our analysis, EEA showed a tendency, although not significant, for superior improvement in visual acuity; which is consistent with previous reports.^12,35^ From an anatomical point of view, we believe that this might correspond to having the possibility to proximally decompress the optic canal from below. Concerning visual fields we found that TCA had a slightly better outcome, but it was not statistically significant. This might be explained due to the early access and dissection to the most dorsal and posterior aspect of the tumor which is in contact with the optic chiasm. Both groups (TCA & EEA) had similar tumor characteristics distribution, not finding differences in the preoperative variables. Due to a small sample size, *p*-values were not as low as desired.

We advise from this study that clinical status of the patient, specifically visual acuity and visual fields, should be specially considered in decision making algorithms since we might see clear advantages of each of these two approaches regarding visual outcome in the postoperative phase. Further research with bigger samples is needed to clarify the impact of different approaches on these clinical aspects.

The ideal approach varies from lesion to lesion, mainly depending on the tumor's location, being the lateral extension a feature which some authors consider a cornerstone when deciding which route should be selected. This is based on the fact that a tumor located inferomedially to the optic nerve is better visualized and accessed from a transnasal perspective rather than a transcranial one. While a tumor located above the optic nerve may be accessible via the transnasal route, the visualization of the upper surface of the nerve and its microvasculature is poor; therefore, it is safer to remove a tumor in this location using a TCA. A tumor located laterally to the optic nerve is usually not considered accessible via the EEA. Also, the surgeon's preferences and expertise are determinants on whether these tumors should be approached via a transnasal or a transcranial route, as surgeons who currently perform extended endoscopic techniques can improve the visualization of anatomical structures that were previously considered inaccessible with traditional endoscopic techniques.

For example, in cases 1 and 2, the surgeon's primary criterion for choosing the TCA was the lateral extension beyond the optic nerves, as a conventional EEA did not provide adequate visualization of the entire tumor. In case 3, the surgeon in charge decided to perform a conventional endoscopic approach since the tumor was medial to the optic nerves, which allowed for proper visualization of the tumor through an EEA. In case 4 an individualized decision-making process was performed, and the surgeon's experience in choosing the approach is an important factor. In this case, we performed an extended EEA despite the tumor having lateral extension. Most surgeons, especially those not familiar with complex endoscopic techniques, would have selected a TCA in such a case, which we do not consider an incorrect option. However, we were able to show that an extended EEA (transtubercular, transplanum, transclival) can allow correct visualization of the lateral boundaries of the tumor with satisfactory postoperative results.

The overall study results should not be superior to a tailored approach for each situation, and we should try to consider as many variables as possible in pursuit of achieving the best results.

Overall, scientific literature suggests that both TCA and EEA are effective options for treating TSM. Both techniques can achieve gross total resection and improvement in visual outcomes. However, the EEA may offer additional advantages in terms of lower complication rates and shorter recovery time. The selection of the appropriate surgical approach should be based on individual patient considerations and specific tumor characteristics, as highlighted in several reviewed studies.

In our country, there are no studies that compare these two surgical approaches in a retrospective or prospective manner. Most of the reported studies are from the American literature and do not necessarily reflect the epidemiological characteristics in Latin America. Therefore, this study was planned to identify factors that influence the visual outcomes of patients with TSM by comparing the described surgical techniques for managing this pathology.

## 5 Conclusion

TSM represent a complex pathology; there is no consensus on the standard surgical approach to be used. Current literature shows comparable results in resection rates and visual outcomes for both approaches. However, EEA seems to offer greater improvement in visual acuity; while TCA provides amelioration of visual fields. The ideal approach should be individualized; taking into account the tumor's volume and invasiveness, as well as the patient's visual complaints.

## Funding

This research did not receive any specific grant from funding agencies in the public, commercial, or not-for-profit sectors.

## CRediT authorship contribution statement

**Ricardo Marian-Magaña:** Writing – review & editing, Writing – original draft, Methodology, Formal analysis, Data curation, Conceptualization. **Marcos V. Sangrador-Deitos:** Writing – review & editing, Writing – original draft, Software, Methodology. **Luis Rodríguez-Hernández:** Writing – review & editing, Writing – original draft, Methodology, Investigation, Data curation, Conceptualization. **Jorge A. Lara-Olivas:** Writing – review & editing, Writing – original draft, Conceptualization. **Germán López-Valencia:** Writing – review & editing, Writing – original draft, Software, Investigation. **Rodolfo Villalobos-Díaz:** Writing – original draft, Investigation, Formal analysis, Conceptualization. **Jorge F. Aragón-Arreola:** Writing – review & editing, Software, Methodology. **Karen E. Padilla-Leal:** Writing – review & editing, Writing – original draft. **Jesús Humberto García-Zazueta:** Writing – review & editing. **Alfredo Camacho-Castro:** Writing – review & editing. **Juan L. Gómez-Amador:** Supervision, Writing – review & editing.

## Declaration of competing interest

The authors declare that they have no known competing financial interests or personal relationships that could have appeared to influence the work reported in this paper.

## Abbreviation list

ACA =: anterior cerebral artery
CNS =: central nervous system
CSF =: cerebrospinal fluid
EEA =: Endoscopic endonasal approaches
ETSS =: expanded transsphenoidal surgery
HM =: hand motion
ICA =: internal carotid artery
LP =: light perception
MRI =: magnetic resonance imaging
NLP =: no light perception
SD =: standard deviation
TCA =: transcranial approaches
TSM =: tuberculum sellae meningiomas
VFD =: visual field deficit
VIS =: visual impairment score
